# An overview of ethical frameworks in public health: can they be supportive in the evaluation of programs to prevent overweight?

**DOI:** 10.1186/1471-2458-10-638

**Published:** 2010-10-22

**Authors:** Marieke ten Have, Inez D de Beaufort, Johan P Mackenbach, Agnes van der Heide

**Affiliations:** 1 Department of Medical Ethics, Erasmus Medical Centre, Dr. Molewaterplein 50, Rotterdam, The Netherlands; 2 Department of Public Health, Erasmus Medical Centre, Dr. Molewaterplein 50, Rotterdam, The Netherlands

## Abstract

**Background:**

The prevention of overweight sometimes raises complex ethical questions. Ethical public health frameworks may be helpful in evaluating programs or policy for overweight prevention. We give an overview of the purpose, form and contents of such public health frameworks and investigate to which extent they are useful for evaluating programs to prevent overweight and/or obesity.

**Methods:**

Our search for frameworks consisted of three steps. Firstly, we asked experts in the field of ethics and public health for the frameworks they were aware of. Secondly, we performed a search in Pubmed. Thirdly, we checked literature references in the articles on frameworks we found. In total, we thus found six ethical frameworks. We assessed the area on which the available ethical frameworks focus, the users they target at, the type of policy or intervention they propose to address, and their aim. Further, we looked at their structure and content, that is, tools for guiding the analytic process, the main ethical principles or values, possible criteria for dealing with ethical conflicts, and the concrete policy issues they are applied to.

**Results:**

All frameworks aim to support public health professionals or policymakers. Most of them provide a set of values or principles that serve as a standard for evaluating policy. Most frameworks articulate both the positive ethical foundations for public health and ethical constraints or concerns. Some frameworks offer analytic tools for guiding the evaluative process. Procedural guidelines and concrete criteria for solving important ethical conflicts in the particular area of the prevention of overweight or obesity are mostly lacking.

**Conclusions:**

Public health ethical frameworks may be supportive in the evaluation of overweight prevention programs or policy, but seem to lack practical guidance to address ethical conflicts in this particular area.

## Background

Is a campaign that stresses the importance of a healthy weight acceptable when it stigmatizes overweight persons? At what point does encouraging physical activity in the workplace become too intrusive in the personal life sphere? Is policy to inform people about health risks of obesity ethically sound when it does not reach people from ethnic minorities? Much public health activity is going on in the field of preventing overweight and obesity. Sometimes this raises pressing ethical questions. Suppose that a public health professional is determined to design a program that will not raise ethical objections from society. Or suppose that he is faced with the question whether to implement a program or not. Or that he must justify a controversial program in the national media. Assuming that this professional did not receive much training in ethics, he may need some guidance in dealing with thorny ethical issues and in articulating the ethical foundations underlying programs to prevent overweight [1]. Where can he turn to?

Analysing ethical issues in public health programs and policy requires a specific field in ethics [2,3]. Public health is generally understood to be 'the science and art of preventing disease, prolonging life and promoting health through the organised efforts of society'[4]. The ethically relevant features of public health differ from those of clinical medicine in at least two respects. First, traditional clinical ethics often addresses the individual relationship between physician and patient, but public health focuses on the population. Second, the emphasis of clinical ethics is predominantly of medical cure and care, whereas public health is mainly concerned with prevention[5,6].

Public health ethics conducts analysis at different levels of abstraction. Not all theories in public health ethics are designed for guiding decision-making in daily practice. According to Dawson, the primary aim of *theories *is to provide *justification *for actions. By contrast, *ethical frameworks *are more concrete instruments that are aimed at assisting professionals in *deliberating *about ethical aspects of programs and policy in order to support the day to day decision-making about their implementation[7].

Several ethical frameworks have been developed for evaluating public health policy. Such frameworks may also be useful in the field of preventing overweight or obesity. The aim of this paper is to give an overview of currently available ethical frameworks that can be useful for evaluating programs to prevent overweight and/or obesity.

## Methods

We identified relevant frameworks by asking 15 experts for the frameworks they were aware of that may be useful in evaluating ethical aspects of public health interventions or prevention of overweight or obesity. 7 experts in the fields of public health ethics, medical ethics and obesity, from various countries, responded. They identified six frameworks [5,8-12]. In order to be as complete as possible, we also searched for frameworks in Pubmed [13]. This search was limited to frameworks that were published in English after 1995. Frameworks that are specifically focussed on public health issues other than overweight and obesity, such as smoking and vaccination, and frameworks for screening programs were excluded [14-21]. The search strategy is described in appendix 1. Literature references in the articles on frameworks we found were also checked. This search provided no additional frameworks.

All papers and documents in which the frameworks were described were scrutinized by one author (MtH) and discussed in detail with two other authors (AvdH and IDdB). We assessed the area on which the available ethical frameworks focus, the users they target at, the type of policy or intervention they propose to address, and their aim. Further, we looked at their structure and content, that is, tools for guiding the analytic process, the main ethical principles or values, possible criteria for dealing with ethical conflicts, and the concrete policy issues they are applied to.

In our analysis we assumed that the practical usefulness of frameworks for evaluating the ethical aspects of programs to prevent overweight and/or obesity is determined by a number of characteristics. To start with, the framework should be applicable to concrete programs for prevention of overweight and/or obesity. Next, according to Dawson's above-mentioned definition of frameworks, it should be practically feasible. Procedural guidelines for applying the framework may help satisfying this criterion. According to the same definition, it should facilitate deliberation about ethical aspects of programs. Also following from Dawson's definition, it should provide criteria for making a decision regarding the acceptability of implementing programs. Furthermore, the framework should map negative as well as positive normative aspects of a program. An ethical evaluation that only pays attention to either ethical strengths or ethical weaknesses would be unbalanced and incomprehensive, which diminishes its practical value. A last characteristic holds that the framework should address all ethical issues that programs to prevent overweight and/or obesity may involve, that is, effectiveness, psychosocial effects, equality, information, liberty, responsibility, privacy and cultural values.

## Results

An overview of several characteristics of the six selected frameworks is presented in table 1. All frameworks address the area of public health in general. The Nuffield framework is the only one that includes a specific section about the ethical issues in prevention of obesity. The Public Health Leadership Society framework [8] and the framework by Childress et al. [10] focus on public health policy in the United States, whereas the Europhen framework [9] concentrates on public health policy in Europe. Tannahill's framework [6] is directed at the area of public health, health promotion and health improvement. In the following section, each of the selected frameworks is shortly described in order of publication. Further details can be found in appendix 2.

**Table 1:** Overview of frameworks

	Kass	**Childress et al**.	Public Health Leadership Society	Europhen	Nuffield	Tannahill
**Title**	An ethics framework for public health	Public health ethics: mapping the terrain	Principles of the ethical practice of public health.	Public policies, law and bioethics: a framework for producing public health policy across the European Union	Public health: ethical issues	Beyond evidence- to ethics: a decision-making framework for health promotion, public health and heath improvement

**Year issued**	2001	2002	2002	2006	2007	2008

**Area**	Public health	Public health in the USA	Public health in the USA	Public health in the EU	Public health	Health promotion, public health and health improvement

**Target group**	Professionals	Public health agents	Institutions with an explicit public health mission	Policymakers in the European Union	Policymakers in government, industry, other organisations and individuals	Decision-makers

**Type of policy or intervention that is addressed**	Interventions, policy proposals, research initiatives, programs	Interventions	Public health practice, including ideals and policies of institutions	Policy	Measures, policy	Policies, programs, services, activities

**Aim**	To indicate ethical implications of programs, to indicate defining values of public health	To provide a rough conceptual map of public health ethics, to help thinking through and resolving conflicts between promoting public health and other moral requirements	To guide institutions by clarifying distinctive elements of public health and the related ethical principles, to provide a standard to which public health institutions can be hold accountable	To help producing common approaches to public health policy across the European Union, especially with regard to tensions between private and public interests	To help considering ethical issues of measures and policy for health improvement	To indicate the function of evidence and ethics in founding policies, to indicate what actions should be implemented

**Analytic tool**	*Six-Step-Questionnaire*	None	None	None	*Intervention-Ladder*	*Decision-Making Triangle*

**Set of principles, values or recommendations**	Values are mentioned in the text, for instance: *public health seeks to improve the well-being of communities*	9 General moral considerations, for instance: *producing benefits*	12 Principles of the ethical practice of public health, for instance: *public health should address principally the fundamental causes of disease and requirements for health, aiming to prevent adverse health outcomes*	11 Recommendations for more effective ways of developing and implementing policy that attracts greater public support, for instance: *public health should strive to create an environment that structures and facilitates individual health, wellbeing and flourishing*	10 principles (Stewardship model), for instance: *acceptable public health goals include reducing the risks of ill health that result from other people's actions, such as drink driving and smoking in public places*	10 possible ethical principles, for instance: *do good*

**Main ethical values**	Well-being Privacy and confidentiality Liberty and self-determination Distributive justice Procedural justice These values have been extracted from the description of the considerations of questions 3, 5 and 6.	Well-being Utility Distributive justice and fairness Procedural justice and participation Liberty and autonomy Privacy and confidentiality Trustworthiness Transparency and openness These values have been extracted from the nine moral considerations that are provided in appendix 2.	Well-being Individual rights Participation Empowerment Equality Evidence based Transparency Effectiveness Consent Swiftness Cultural value pluralism Respect for environment Confidentiality and privacy Professionalism Trustworthiness These values have been extracted from the twelve principles that are provided in appendix 2.	Well-being Empowerment Individual rights Liberty and autonomy Personal responsibility and duties Communitarianism Participation Transparency Accountability Trust Confidentiality and privacy Swiftness These values have been extracted from the eleven recommendations that are provided in appendix 2.	Well-being Care of the vulnerable Empowerment Autonomy Fairness and equality Liberty and self determination Openness Privacy These values have been extracted from the ten principles that are provided in appendix 2.	Well-being Equity Respect Empowerment Sustainability Social responsibility Participation Openness Accountability These values have been extracted from the ten ethical principles that are provided in appendix 2.

**Criteria for dealing with ethical conflict**	-The greater the burden, the greater must be the expected public health benefit. -The more uneven the benefits and burdens are divided between groups, the greater must be the expected benefit. -Coercive programs should be kept to a minimum, should never be implemented when a less restrictive program would achieve comparable goals, and should be implemented only in the face of a clear public health need and good data demonstrating effectiveness. Disagreements about balancing burdens and benefits should be solved through a system of fair procedures that require a democratic process, including public hearings to consider minority views.	Within particular circumstances promoting the goals of public health (producing benefits, preventing harms and producing utility) may override other moral considerations (such as individual liberty or justice), provided that the following justificatory conditions are met: -Effectiveness -Proportionality -Necessity -Least infringement -Public justification Dealing with conflicts in a fair and trustworthy manner requires a process of public accountability. This involves soliciting input from the relevant publics during the formulation of public health policies as well as justifying to the relevant publics what is being undertaken after decisions have been made.	Not specified	Not specified	-The overall aim should be to achieve the desired health outcomes while minimising restrictions on people's freedom. -The more intrusive a program is, the more benefits its must create. -Ideally the principles should not be infringed, and when infringement is deemed necessary sound justification is required. -The classical harm principle, care of the vulnerable, autonomy and consent are of special importance, either because infringing them can have significant consequences, or because they are of particular relevance to public health interventions.	Documenting judgements can be of value both in consultation and in continuing constructive dialogue after decisions have been made. In case of disagreement, those who disagree may understand what decisions were based on and can argue for a different decision based on the same principles.

**Application to concrete policy issues**	Yes (that is to avian influenza preparedness)	Yes (that is to screening programs)	No	Yes (that is to a smacking ban, regulation regarding wearing car seat belts, legalising cannabis, water fluoridation, compulsory immunization, smoking ban in public places)	Yes (that is to infectious disease, obesity, alcohol and smoking and fluoridation of water)	No

### Kass: An ethics framework for public health [5,22]

Kass aims to raise awareness of the ethical issues of proposed programs and to help consider means of responding to them. Her framework includes an analytic tool that consists of a step-by-step-list of six questions for deciding how the burdens and benefits of an intervention can be fairly balanced (see table 2), and a description of relevant ethical considerations. The framework expresses the defining values of public health, including positive obligations to improve population health and to reduce social inequalities. Kass further distinguishes three categories of ethical burdens, namely: risks to privacy and confidentiality, risks to liberty and self-determination, and risks to justice. She describes specific burdens for six types of public health activities, two of which are used in overweight prevention. The first type of activities, health education, is relatively unproblematic since it is voluntary and aimed at empowerment, but may nevertheless give rise to ethical problems: lack of effectiveness; manipulation, coercion and inadequate information; paternalism; stigmatization resulting from targeting; and directing personal choice by using incentives. The second type of activities, regulations and legislation, are considered the most intrusive approach to public health: by imposing penalties for non-compliance they threaten liberty and self-governance; they may involve health risks (for instance in case of vaccination); and if they pose undue burdens on particular segments of society they can be unjust. A number of criteria should help weighing burdens and benefits. First, the greater a program's ethical burden, the greater its expected public health benefit must be. Second, the more uneven the benefits and burdens are divided between groups, the greater the expected benefit must be. And third, coercive programs must be kept to a minimum. Within a pluralistic society, the balancing of benefits and burdens will inevitably lead to disagreements. They should be solved through a system of fair procedures. This requires a democratic process including public hearings to consider minority views.

**Table 2:** Ethical framework for public health by Kass [5]

1.	What are the public health goals of the proposed program?
2.	How effective is the program in achieving its stated goals?
3.	What are the known or potential burdens* of the program?
4.	Can burdens be minimised? Are there alternative approaches?
5.	Is the program implemented fairly?**
6.	How can the benefits and burdens of a program be fairly balanced?

*Burdens refer to risks for privacy and confidentiality, liberty and self-determination, and justice.

**Fair implementation refers to the ethical principle of distributive justice.

### Childress et al.: Public Health Ethics [8]

Childress et al. provide a conceptual map of public health ethics in the United States. Furthermore, they attempt to resolve conflicts between the promotion of public health and other moral values. The framework consists of nine general moral considerations in public health ethics (see table 3). When these principles conflict with each other, each may have to yield in some circumstances, because they have no absolute character and are not hierarchically ordered. The first three considerations reflect the goals of public health: producing benefits, preventing harms, and maximizing the balance of benefits over harms and costs. Under certain conditions these public health goals may override the other six moral considerations, such as justice, liberty and privacy. Those conditions involve that (1) the program is *effective *in protecting public health; (2) its benefits to public health outweigh the infringement of moral considerations (*proportionality*); (3) there is no alternative program that is less morally troubling (*necessity*); (4) the degree to which the program is infringing should be minimised (*least infringement*); and (5) public health agents should explain and justify the infringement (*public justification*). Additionally, the process of resolving conflicts between public health goals and other moral considerations must be transparent. Transparency involves honestly disclosing information, but also seeking information by consulting the public.

**Table 3:** General Moral Considerations of public health ethics by Childress [8]

• producing benefits
• avoiding, preventing and removing harms
• producing the maximal balance of benefits over harms and other costs (often called utility)
• distributing benefits and burdens fairly (distributive justice) and ensuring public participation, including the participation of affected parties (procedural justice)
• respecting autonomous choices and actions, including liberty of action
• protecting privacy and confidentiality
• keeping promises and commitments
• disclosing information as well as speaking honestly and truthfully (often grouped under transparency)
• building and maintaining trust

Childress et al. furthermore provide criteria for defining the degree of paternalism of public health interventions. Coercive intervention in behaviour that is voluntary and that affects primarily the actor himself is called strong paternalism and is difficult to justify.

### Public Health Leadership Society [2,9,23]

The Public Health Leadership Society's *Principles of the ethical practice of public health *is a code of ethics for public health institutions. It was proposed in 2001 and adopted by several organizations such as the American Public Health Association. It serves both as a guide for public health institutions and as a standard to which these institutions can be hold accountable. The framework consists of a set of twelve ethical principles (see table 4 for a selection of principles, the complete set can be found in appendix 2). The principles are related to the ten essential public health services. For instance, the principle of 'collaboration' is linked to the public health service 'mobilize community partnerships to identify and solve health problems'. One of the key beliefs underlying the framework is the notion of 'interdependence' between humans. This means that each person both affects and depends upon others. It relates to public health's concern with the population instead of individuals. The idea of interdependence serves to correct a perspective that is only concerned with the individual right to autonomy. The framework is not designed as an instrument for resolving particular conflicts. Instead it provides an overview of principles that should be considered in a dispute.

**Table 4:** Sample of principles by PHLS[9]

1.	Public health should address principally the fundamental causes of disease and requirements for health, aiming to prevent adverse health outcomes.
2.	Public health should achieve community health in a way that respects the rights of individuals in the community.
3.	Public health policies, programs and priorities should be developed and evaluated through processes that ensure an opportunity for input from community members.
4.	Public health should advocate and work for the empowerment of disenfranchised community members, aiming to ensure that that the basic resources and conditions necessary for health are accessible to all.

### Europhen [10]

Europhen is directed at producing common approaches to public health policy across the European Union. The framework does not contain an analytic tool, or a set of principles or values. Instead, it examines normative issues that should guide public health programs and their implementation. The Europhen report firstly provides a theoretical analysis of tensions between private and public interests. Secondly, it compares public health structures and policy responses to selected public health problems (not including overweight and obesity) in member states of the European Union. Thirdly, it offers an empirical analysis of public attitudes regarding public versus private interests for a number of topics, such as parental rights, incentives and enforcement, solidarity, rights and responsibilities. European policy for public health should be pluralistic and flexible, because the variety of socio-economic settings in individual countries will lead to different priorities. The report proposes three main policy goals: promotion of population health, promotion of health-related autonomy and promotion of health-related equality. Furthermore eleven recommendations are made for more effective ways of developing and implementing policy that attracts greater public support (see table 5 for a sample and appendix 2 for the complete set). Public health should, for instance, 'strive to create an environment and structures that facilitate individual health, wellbeing and flourishing'.

**Table 5:** Sample of policy recommendations by Europhen[10]

• Public health should strive to create an environment that structures and facilitates individual health, wellbeing and flourishing.
• Public health has a strong role to play in ensuring that people feel part of a society so that they can make a contribution to society.
• Public health institutions should respect the confidentiality of information that can bring harm to an individual or community if made public.
• Where there are risks to health, public health institutions should act in a timely manner on the information available.

### Nuffield Council on Bioethics[11]

The Nuffield Council on Bioethics aims to help considering the ethical issues of public health policy. It offers two analytic tools, the 'stewardship model' and the 'intervention ladder'. The stewardship model describes acceptable goals and restrictions for public health policy. It departs from the position that the state has a duty to enable people to lead healthy lives. Next to this, governments should try to remove inequalities that affect disadvantaged groups or individuals. Acceptable public health goals include for example "reducing the risks of ill health that result from other people's actions". Restrictions include "coercing adults to lead healthy lives". The principles of the stewardship model are not listed in an order of priority. The overall aim should be to achieve the desired health outcomes while minimising restrictions on people's freedom. Furthermore, special attention should be paid to consent and care of the vulnerable. The 'Intervention ladder' lists levels of intrusiveness of public health policies, from "do nothing" until "eliminate choice" (see table 6). The higher upon the ladder a program is, the stronger its justification needs to be.

**Table 6:** Intervention ladder by Nuffield Council on Bioethics[11]

• Eliminate choice
• Restrict choice
• Guide choice through disincentives
• Guide choice through incentives
• Guide choices through changing the default policy
• Enable choice
• Provide information
• Do nothing or simply monitor the current situation

The report includes, by means of example, a case study on ethically sensitive issues in obesity prevention. It provides policy recommendations on obesogenic environments; food labelling; protecting children; personal responsibility and NHS treatment, the roles of the food and drink industries, the government and public services; collecting data about childhood obesity and intervention in the home for childhood obesity. One of its conclusions is for instance that 'There is an ethical justification for the state to intervene in schools to achieve a more positive attitude towards healthy eating, cooking and physical activity'.

### Tannahill: Beyond evidence - to ethics[12]

Tannahill's framework describes the position of evidence and ethics in decision-making about public health interventions. Using the framework should lead to a decision whether or not to implement an intervention. The framework consists of a 'decision-making triangle' that has on its top *ten ethical principles*, and *evidence *and *theory *on its bottom (see Table 7). The triangle illustrates Tannahills claim that the emphasis in decision-making should be on the explicit application of an identified set of ethical principles. Available evidence, which is always incomplete, and plausible theory on effectiveness should inform whether a program satisfies the ethical principles. Within this framework the effectiveness of an intervention is essential, but only because it serves the ethical principles. The set of principles includes for instance social responsibility and sustainability. How the principles should be interpreted and weighed, depends upon political and cultural perspectives. In case of disagreement, documenting judgements should facilitate a constructive dialogue. An explicit use of the triangle is supposed to contribute to the values of openness and accountability.

**Table 7:** Decision-making triangle by Tannahill[12]

## Discussion

Our overview of ethical frameworks shows that various efforts have been made to help policymakers and public health professionals deliberating about the ethical aspects of public health policy and programs. Kass offers a step-by-step procedure to weigh the burdens and benefits of a program [5]. Childress et al. assist in evaluating programs that promote public health but that infringe upon other moral considerations[8]. PHLS provides ethical standards to guide the practices of American public health institutions[9]. Europhen gives insight in ethically relevant public health differences within the European Union and in ways to bridge them[10]. The Nuffield stewardship model distinguishes acceptable goals and restrictions of public health programs, and its intervention ladder helps in balancing a program's benefits and its intrusion in people's lives[11]. Finally, Tannahill's triangle assists in integrating ethics and evidence in such a deliberation[12].

However, all frameworks have limitations with respect to their practical value in the evaluation of programs to prevent overweight and/or obesity (see Table 1). Nuffield is the only framework that specifically addresses obesity prevention[11]. Four frameworks can be applied to concrete programs related to overweight or other public health problems, but Europhen and PHLS cover a more abstract question, namely: 'what ethical values should direct public health policy?'[9,10]

We found it remarkable that none of the frameworks specifies when and by who it should be used. This may stem from the desire to develop a framework that is broadly applicable and that can be used by anybody at anytime. We think that users of a framework would benefit from procedural guidelines for applying the framework. Especially professionals who have no experience with ethical consultation and who must fit the application of an ethical framework into their other tasks may profit from suggestions. Advice about the best time to apply a framework (before the implementation of a program or already during the designing phase) and about the number and background of the persons who are to use it, may save efforts and thus lower the threshold of using a framework.

Kass, Nuffield and Tannahill offer an *analytic tool*, which is an instrument to guide the evaluative process. These tools comprise a decision-making-triangle, a step-by-step-questionnaire and a ladder to indicate proportionality[5,11,12]. Such tools make a framework more practically useful for policymakers than merely a set of ethical values does. In addition, framing questions may contribute more to adequate deliberation of the ethical aspects of programs than providing fixed answers or guidelines. The Europhen policy recommendations, for instance, aim to help policymakers solving ethical issues by indicating the direction that policy should take[10,11]. As opposed to this, Kass and Tannahill for instance frame the *questions *that should be raised and thereby encourage the process of deliberation. Kass leaves answering the question 'How can burdens and benefits be fairly balanced?' up to the public health professional or policymaker[5]. Tannahill's triangle formulates the steps that are to be taken in the process of deliberation without filling in the decisions that should be made[12].

No simple solution seems to be available for dealing with ethical conflicts, although it is precisely the tendency of ethical principles to infringe upon each other that creates the need for frameworks. The designers of the frameworks agree that the principles cannot be ordered according to priority but must be weighed in concrete circumstances. Kass, Nuffield and Childress et al. identify criteria for this weighing process[5,8,11]. They agree on the fact that the burdens of a public health program should be in proportion to its benefits. Furthermore they refer to the 'harm principle', which implies that restrictions to people's freedom should be minimized and that they are only justified in case of a clear public health requirement. Childress et al. distinguish themselves from the other frameworks by putting ethical conflicts at the centre, rather than merely pointing out ethical values[8]. They point out five justificatory conditions for public health programs that infringe moral principles, namely: effectiveness, proportionality, necessity, least infringement, and public justification. PHLS and Europhen do not articulate criteria for dealing with ethical conflicts[9,10].

However, even with sound weighing criteria, disagreement about the outcome of a framework is inevitable. That is because personal, cultural and political perspectives affect the process of interpretation and weighing. Several frameworks recommend fair procedures for dealing with difference of opinion. Tannahill encourages an explicit use of the decision-making triangle, including documenting judgements. This may contribute to consultation and dialogue, and enables a discussion about disagreements on the basis of shared principles[12]. Kass argues for a democratic process and public hearings to consider minority views[5]. And Childress et al., to conclude, advocate a transparent process for expressing justice and sustaining public trust. Such a process requires both asking input from the public, as well as offering justifications for decisions that have been made[8].

Most of the frameworks aspire not only to set ethical boundaries (such as restrictions to interference), but also to articulate positive ethical foundations for public health (such as the duty to diminish inequalities), which seems to contribute to their practical value. However, the usefulness for prevention of overweight or obesity requires that all ethical issues that are relevant for this field are clearly addressed. The majority of the frameworks frames abstract ethical values without outlining the concrete ethical issues they may give rise to. Most frameworks contain a set of ethical values. Some are articulated as principles, whereas others take the form of policy recommendations or goals. Only Kass' framework does not include a list of values, but her description of relevant ethical considerations does refer to them[5]. These abstract ethical values do more or less cover the relevant ethical themes. For instance, the issues of *liberty *and *responsibility *that may occur in programs to prevent overweight are in all frameworks covered by the classical values of liberty and responsibility. Nuffield, Europhen, PHLS, and Tannahill explicitly mention social responsibility and stress the need for creating a healthy environment and facilitating healthy behaviour, which are both relevant for the prevention of overweight[9-12]. Europhen is the only framework that emphasizes that citizens also have duties, thereby paying attention to the debate about accountability for an unhealthy weight. It states that 'citizens consider themselves as consumers of healthcare who see health services as their right as taxpayers. However rights have reciprocal responsibilities, and the public must be reminded of these.'[10]

Furthermore, all frameworks (except for Tannahill's) address the issues of *privacy *by mentioning the values of privacy and confidentiality. And all frameworks address the issue that the *effectiveness *of a program to prevent overweight may be uncertain or unfavourable by mentioning the values of well-being, and sometimes by mentioning the value of utility (producing the maximal balance of benefits over harms and other costs)[5,8,11,12].

However, almost none of the frameworks describes the concrete ethical issues that may occur in programs. The issue of *equality *is covered in all frameworks except for the recommendations by Europhen[10]. But knowing that equality is an important value does not specify that programs to prevent overweight may increase already existing health inequalities by being least effective among groups that have the highest risk of developing overweight. Likewise, the importance of providing adequate *information *is covered by the values of autonomy, transparency and trustworthiness that are mentioned in all frameworks. However, inadequate information is sometimes distributed by accident, and the frameworks do not provide guidelines about what adequate information exactly entails and how to prevent the accidental distribution of inadequate information.

Furthermore, two issues were absent in most frameworks. One issue, that interference may occur with cultural and social values of food and eating habits, is only covered by the PHLS framework, which articulates the need to respect cultural value pluralism: 'Public health programs and policies should incorporate a variety of approaches that anticipate and respect diverse values, beliefs and cultures within the community.'[9] The other issue, namely the potential negative *psychosocial consequences *of programs to prevent overweight (such as uncertainty, fear and weight concerns about the health risks of overweight and obesity, stigmatization and blaming, and unjust discrimination), is by most frameworks only covered to a limited extent. Only Kass and Nuffield warn against the potentially stigmatizing effects of targeted messages[5,11]. None of the frameworks goes into detail about how programs can reinforce the negative image of overweight people, how they may create unnecessary concerns about health risks, or how they may undermine self-confidence for people who do not succeed in losing weight. The lack of attention for cultural values, and for stigmatization and other psychosocial issues may be explained by the fact that these issues are particularly relevant for the field of overweight prevention and less for other fields in public health.

Designers of frameworks face the challenge of acknowledging the complex character of ethical issues, without loosing sight of their main task, namely guiding professionals in the process of articulating and dealing with ethical issues. Presenting a set of abstract ethical principles does not provide guidance to policymakers who are not familiar with ethics. This is not a shortcoming of the frameworks in themselves, since each has its own particular aims, but it does indicate that our last criterion is not satisfied by the available frameworks. Thus, it is questionable to what extent the frameworks facilitate deliberation among policymakers regarding the concrete ethical issues in the prevention of overweight and obesity.

Our study has several limitations. It is possible that we overlooked one or more frameworks that are suitable for evaluating the ethical aspects of programs to prevent overweight and/or obesity. Furthermore, our analysis of the usefulness of frameworks is restricted to self-developed criteria. We did not interview policymakers in the field of overweight prevention about the usefulness of frameworks and we did not test the frameworks on actual programs.

## Conclusions

We found no framework that takes into account all ethical issues that are relevant for the prevention of overweight. Further, the practical value of currently available frameworks is limited in several aspects. Practically valuable frameworks that address all relevant ethical issues are needed because much public health activity is going on in the field of preventing overweight that has distinct ethical features, such as the issue of stigmatization of behaviour.

## Competing interests

The authors declare that they have no competing interests.

## Authors' contributions

MtH performed the literature search, scrutinized the papers and documents in which the papers were subscribed and drafted the manuscript. AvdH and IDdB participated in a detailed discussion about the frameworks and helped to draft the manuscript. JPM conceived of the study, participated in its design and coordination and revised the manuscript. All authors read and approved the final manuscript.

## Appendix 1. Search strategy in pubmed

(((ethic*[ti] OR moral[ti] OR normative[ti]) AND ("decision making"[ti] OR framework*[ti] OR guideline*[ti] OR principle*[ti] OR code*[ti])) OR (("ethical decision making" OR "ethical framework" OR "ethics framework" OR "ethical guideline" OR "ethical guidelines" OR "ethics guidelines" OR "ethical principle" OR "ethics principle" OR "ethical principles" OR "ethics principles" OR "ethical code" OR "ethics code" OR "ethical codes" OR "ethics codes" OR "moral framework" OR "normative framework" OR "moral guidelines" OR "normative guidelines" OR "moral principle" OR "normative principle" OR "moral principles" OR "normative principles" OR "moral code" OR "moral codes") AND ("guideline"[Publication Type] OR "guidelines as topic"[MeSH Terms]))) AND ("public health" OR "public health"[mesh:noexp] OR "public health practice"[mesh]) AND 1995:3000[dp] AND eng[la] [11]

## Appendix 2. Overview of principles and values in the frameworks

Set of ethical principles, values or recommendations

Kass

Instead of a set of principles or recommendations, values are mentioned in the text

**Childress et al**.

General moral considerations

-producing benefits

-avoiding, preventing and removing harms

-producing the maximal balance of benefits over harms and other costs (often called utility)

-distributing benefits and burdens fairly (distributive justice) and ensuring public participation, including the participation of affected parties (procedural justice)

-respecting autonomous choices and actions, including liberty of action

-protecting privacy and confidentiality

-keeping promises and commitments

-disclosing information as well as speaking honestly and truthfully (often grouped under transparency) and

-building and maintaining trust

Public Health Leadership Society

Principles of the ethical practice of public health

1. Public health should address principally the fundamental causes of disease and requirements for health, aiming to prevent adverse health outcomes.

2. Public health should achieve community health in a way that respects the rights of individuals in the community.

3. Public health policies, programs and priorities should be developed and evaluated through processes that ensure an opportunity for input from community members.

4. Public health should advocate and work for the empowerment of disenfranchised community members, aiming to ensure that that the basic resources and conditions necessary for health are accessible to all.

5. Public health should seek the information needed to implement effective policies and programs that protect and promote health.

6. Public health institutions should provide communities with the information they have that is needed for decisions on policies or programs and should obtain the community's consent for their implementation.

7. Public health institutions should act in a timely manner on the information they have within the resources and the mandate given to them by the public.

8. Public health programs and policies should incorporate a variety of approaches that anticipate and respect diverse values, beliefs and cultures in the community.

9. Public health programs and policies should be implemented in a manner that most enhances the physical and social environment.

10. Public health institutions should protect the confidentiality of information that can bring harm to an individual or community if made public. Exceptions must be justified on the basis of the high likelihood of significant harm to the individual or others.

11. Public health institutions should ensure the professional competence of their employees.

12. Public health institutions and their employees should engage in collaborations and affiliations in ways that build the public's trust and the institution's effectiveness.

Europhen

Recommendations for more effective ways of developing and implementing policy that attracts greater public support

1. Public health should strive to create an environment that structures and facilitates individual health, wellbeing and flourishing.

2. Public health should achieve population health in a way that respects the rights of individuals.

3. Public health policies must take heed of the pre-eminence of autonomy in European society.

4. Citizens consider themselves as consumers of healthcare who see health services as their right as taxpayers. However rights have reciprocal responsibilities, and the public must be reminded of these.

5. Public health has a strong role to play in ensuring that people feel part of a society so that they can make a contribution to society.

6. The public are unlikely to support policies which they do not understand or which they see as unconnected to their lives.

7. Public health policy should be implemented in a transparent manner that facilitates accountability.

8. There is a need to actively build trust in public health policy.

9. A balanced approach is required between incentives and restrictions.

10. Public health institutions should respect the confidentiality of information that can bring harm to an individual or community if made public.

11. Where there are risks to health, public health institutions should act in a timely manner on the information available.

Nuffield

The Stewardship model

Acceptable public health goals include:

-reducing the risks of ill health that result from other people's actions, such as drink-driving and smoking in public places;

-reducing causes of ill-health relating to environmental conditions, for instance provision of clean drinking water and setting housing standards;

-protecting and promoting the health of children and other vulnerable people;

-helping people to overcome addictions that are harmful to health or helping them to avoid unhealthy behaviours;

-ensuring that it is easy for people to lead a healthy life, for example by providing convenient and safe opportunities for exercise;

-ensuring that people have appropriate access to medical services; and

-reducing unfair health inequalities.

At the same time, public health programs should:

-not attempt to coerce adults to lead healthy lives;

-minimise the use of measures that are implemented without consulting people (either individually or using democratic procedures); and

-minimize measures that are very intrusive or conflict with important aspects of personal life, such as privacy

Tannahill

Ten possible ethical principles for health promotion, public health and health improvement

Do good

Do not harm

Equity

Respect

Empowerment

Sustainability

Social responsibility

Participation

Openness

Accountability

## Pre-publication history

The pre-publication history for this paper can be accessed here:

http://www.biomedcentral.com/1471-2458/10/638/prepub

## References

[B1] Mackie P Sim F The ethics of public health decision making Public Health 2004 118 311 312 10.1016/j.puhe.2004.05.001 15178136

[B2] Thomas JC Sage M Dillenberg J Guillory JV A code of ethics for public health American Journl of Public Health 2002 92 7 1057 1059 10.2105/AJPH.92.7.1057 PMC1447186 12084677

[B3] Sindall C Does health promotion need a code of ethics? Health Promotion International 2002 17 3 201 203 10.1093/heapro/17.3.201 12147634

[B4] Acheson R Public health in England: the report of the committee of the enquiry into the future development of the public health function 1988 HMSO. London

[B5] Kass NE An ethics framework for public health Am J Public Health 2001 91 11 1776 1782 10.2105/AJPH.91.11.1776 11684600 PMC1446875

[B6] Dawson A Verweij M Ethics, prevention and public health 2007 Oxford: Clarendon Press

[B7] Dawson A Peckham S, Hann A Theory and practice in public health ethics: a complex relationship Public Health Ethics and Practice 2009 Bristol: Policy Press 191 209

[B8] Childress JF Faden RR Gaare RD Gostin LO Kahn J Bonnie RJ Kass NE Mastroianni AC Moreno JD Nieburg P Public health ethics: mapping the terrain J Law Med Ethics 2002 30 2 170 178 10.1111/j.1748-720X.2002.tb00384.x 12066595

[B9] Public Health Leadership Society Principles of the ethical practice of public health. 2.2 edition. USA 2002 11

[B10] Public policies law and bioethics: a framework for producing public health policy across the European Union 2006 European Public Health Ethics Network

[B11] Lord Krebs K Unwin J Public health: ethical issues 2007 London: Nuffield Council on Bioethics

[B12] Tannahill A Beyond evidence--to ethics: a decision-making framework for health promotion, public health and health improvement Health Promot Int 2008 23 4 380 390 10.1093/heapro/dan032 18971394

[B13] Pubmed http://www.ncbi.nlm.nih.gov/pubmed/

[B14] Wilson JMG Jungner G Principles and practice of screening for disease 1968 34 Geneva: World Health Organization

[B15] Fox BJ Framing tobacco control efforts within an ethical context Tob Control 2005 14 Suppl 2 ii38 44 10.1136/tc.2004.008300 16046701 PMC1766190

[B16] Upshur REG Principles for the justification of public health intervention Canadian Journal of Public Health 2002 93 2 101 103 10.1007/BF03404547 PMC6979585 11968179

[B17] Harris J Bortolotti L Irving L An ethical framework for stem cell research in the European Union Health Care Anal 2005 13 3 157 162 10.1007/s10728-005-6439-7 16223207

[B18] Thompson AK Faith K Gibson JL Upshur REG Pandemic influenza preparedness: an ethical framework to guide decision-making BMC Medical Ethics 2006 7 12 17144926 10.1186/1472-6939-7-12 PMC1698926

[B19] Daniels N Accountablity for reasonableness BMJ 2009 321 1300 1301 10.1136/bmj.321.7272.1300 PMC1119050 11090498

[B20] Daniels N Teagarden JR Sabin JE An ethical template for pharmacy benefits Health Aff (Millwood) 2003 22 1 125 137 10.1377/hlthaff.22.1.125 12528844

[B21] Gostin LO Influenza pandemic preparedness: legal and ethical dimensions Hastings Cent Rep 2004 34 5 10 11 10.2307/3527583 15553391

[B22] Kass NE An ethics framework for public health and avian influenza pandemic preparedness Yale J Biol Med 2005 78 5 239 254 17132331 PMC2259154

[B23] Stefanak M Frisch L Palmer-Fernandez G An organizational code of public health ethics: practical applications and benefits Public Health Reports 2007 122 548 551 17639659 10.1177/003335490712200417 PMC1888506

